# Fathers and mothers’ parenting stress and adolescent depressive symptoms: the mediating roles of overt and covert coparenting conflict behaviors

**DOI:** 10.1186/s13034-022-00531-5

**Published:** 2022-12-14

**Authors:** Yizhen Ren, Shengqi Zou, Hui Wang, Jiefeng Ying, Xinyi Wang, Xinchun Wu

**Affiliations:** 1 Beijing Key Laboratory of Applied Experimental Psychology, Faculty of Psychology, Beijing Normal University, Xinjiekouwai Road, Haidian District, Beijing, 100875 China; 2 Center for Mind & Brain Science, Cognition and Hunan Behavior Key Laboratory of Human Province, Department of Psychology, Hunan Normal University, Changsha, 410006 China; 3 Department of Psychology, Faculty of Arts and Sciences, Beijing Normal University at Zhuhai, Zhuhai, 519087 China

**Keywords:** Parenting stress, Adolescent depressive symptoms, Overt and covert coparenting conflict behavior, Interdependent approach

## Abstract

**Background:**

It is yet to be clarified if and how parenting stress was linked to adolescent depressive symptoms during the pandemic.

**Objectives:**

This study adopted an interdependent approach to examine the relationship between parenting stress and adolescent depressive symptoms in Chinese families. It then examined the mediating effects of overt and covert coparenting conflict behaviors.

**Methods:**

As a national survey, data were obtained from different regions in China. Fathers, mothers, and adolescents from 1031 families participated in this study. The fathers and mothers reported parenting stress; the adolescents rated their fathers and mothers’ overt and covert coparenting conflict behaviors and their own depressive symptoms.

**Results:**

Maternal parenting stress was related to adolescent depressive symptoms through the mediating effects of paternal overt and maternal covert coparenting conflict behaviors. Both paternal and maternal parenting stress were directly related to adolescent depressive symptoms. However, maternal parenting stress had a more substantial effect on adolescent depressive symptoms than paternal parenting stress.

**Conclusions:**

The findings support the effects of parenting stress on adolescent depressive symptoms. The study also highlights the mediating roles of paternal overt and maternal covert coparenting conflict behaviors in relationships.

## Introduction

Due to their developmental characteristics, adolescents are particularly vulnerable to depressive symptoms (DS) [19, 25, 51, 68]. Family and parent factors have been shown to play critical roles in the development of child DS [4, 60, 67]. Parental parenting stress (PS) has been proposed as a significant determinant of adolescent adjustment in the parental stress model [1]. Parental PS has been shown to undermine coparenting, which serves as the executive system in the family [6, 17, 52], leading to parental coparenting conflict behaviors (CCB) and eventually resulting in children’s psychological maladjustment [59]. The current study adopts an interdependent approach to examine the relationship between parental PS and adolescent DS. It then investigates the mediating effects of parental CCB in relationships.

### The relationship between parental stress and adolescent DS

While going through home quarantine during the COVID-19 pandemic, adolescents displayed varying degrees of DS [19, 25, 51, 68]. Despite their growing independence, adolescents are still inevitably affected by their families. One such significant family factor is parental PS based on the parental stress model, which refers to the form of stress specific to the role of parents when they perceive that the demands on them exceed their capacity to meet them [1, 6, 14, 48, 54, 60]. Parents have been shown to have experienced elevated levels of PS due to the profound changes brought by the pandemic [7–9, 23, 26]. Parents with excessive parenting stress may experience adverse psychological reactions such as negative perceptions and feelings about their parental role [6, 14, 48, 54, 60]. Such stressful, emotional states and associated negative experiences from parental PS may be conveyed to children and result in negative emotions [6, 14, 48, 54, 60]. Research has established a unique link, both cross-sectionally [48, 54] and longitudinally [60], between parental PS and child emotional problems. During the home quarantine period, adolescents stayed with their parents all day, which may have made them more susceptible to emotional problems [22]. Under such circumstances, this study aims to investigate the relationship between parental PS and adolescent DS during the pandemic.

### The mediating effects of parental overt and covert CCB

In the parental stress model, parental PS can indirectly affect child adjustment in addition to its direct influence [1, 2]. The current study focuses on coparenting as a potential mediating mechanism in the relationship between parental PS and adolescent DS. Coparenting refers to the way parents coordinate with each other in their role as parents [31, 39, 42, 43, 73]. In a parenting alliance, CCB represents the significant mechanism whereby individual parental characteristics (parental PS) exert influence on a child’s psychological adjustments [21, 22]. Previous researchers have found significant mediating effects of other individual parenting behaviors, such as parental involvement, on the effects of parental PS on child adjustment [36, 37]. However, little is known about the mediating effects of parental CCB, as distinguished from other individual parenting behaviors.

Notably, previous research has mainly investigated coparenting relationship quality while neglecting individual CCB in the process [6, 17, 52]. Although fathers and mothers share similar coparenting relationships, they may demonstrate distinct CCB in the coparenting process, which needs further investigation [41, 55, 74]. Parental CCB has been constructed to include two dimensions, namely overt and covert CCB [41, 55, 74]. The overt CCB dimension measures parental conflict behaviors, such as disagreements about child-rearing issues when both parents are present in front of the child. The covert CCB dimension measures undermining and disparaging behavior toward the second parent’s authority or credibility in the absence of the first parent [41, 55, 74]. However, few studies have adopted this innovative approach to measuring parental CCB. Researchers who only measure overt CCB have seldom considered the conflict processes that occur when a parent is alone with the child [29, 31, 32]. This may lead us to miss important information on how such coparenting conflict processes become evident to the child in the absence of the other parent. Therefore, it would be meaningful to assess adolescent perceptions of overt and covert parental CCB and explore the relative influence of various parental CCBs for adolescent psychological adjustments.

Further, there may be differences in the mediating effects of CCB between fathers and mothers, which also requires further exploration. Previous research has usually only included mothers or treated fathers and mothers as a whole [5, 52]. However, paternal and maternal overt and covert CCB may represent different meanings for adolescents and thus have different influences on their psychological adjustments [74]. Indeed, a recent study found that only paternal overt and maternal covert CCB were significantly related to adolescent adjustments [74]. This indicates the necessity and significance of including mothers and fathers simultaneously to investigate the mediating effects of overt and covert CCB in the relationship between parental PS and adolescent DS [61].

Parental PS has been proposed as a significant determinant of coparenting behaviors in the ecological model of coparenting [21]. Parental PS has been shown to act as a crucial factor in influencing individual parent behaviors and child outcomes [6, 11, 20, 24, 37, 66]. Literature has demonstrated that parenting PS can significantly predict negative parenting styles and less parental involvement behavior [6, 37, 56, 66]. However, little research has examined the effects of parental PS on coparenting behaviors. One recent study showed that parental PS could adversely affect overall coparenting quality [52]. A previous study showed that parental PS could lead to lower supportive coparenting relationships [5]. However, these studies neglected individual parental conflict behaviors in the coparenting process. Therefore, little known is known whether overt and covert CCB demonstrates different mediating effects in the relationship between parental PS and adolescent DS.

### Inclusion of both fathers and mothers

**Fig. 1:**
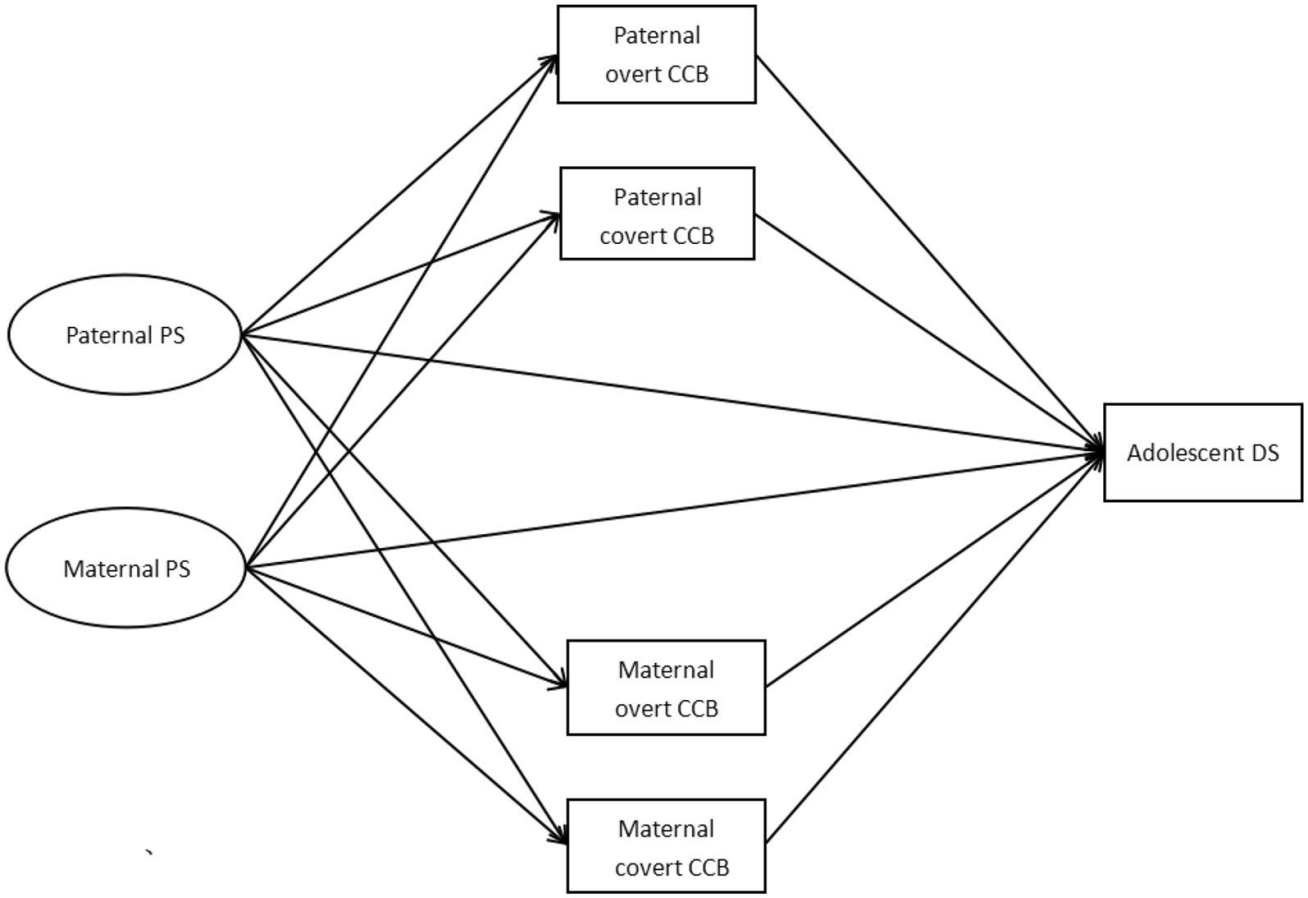
The hypothesized mediation model

According to family system theory, the effect formulated in one subsystem or individual can be directly transferred to another [27]. Two processes derived from it can be used to explain interrelationships among family members. According to the spillover hypothesis, one family member’s experiences or feelings can be transferred among different family subsystems [73], representing the intrapersonal process. Thus, paternal and maternal PS may be associated with each parent’s CCB. The interpersonal process, reflected in the crossover hypothesis, represents the transfer of one family member’s experiences to another [44]. Thus, paternal and maternal PS may not only be associated with each parent’s own CCB but also with their partner’s CCB. Only one study has simultaneously included both fathers and mothers to test the relationship between parental PS and coparenting. The study found that mothers’ PS was related to both parents’ reports of coparenting conflict. But fathers’ PS was only associated with their own coparenting conflict [65]. Therefore, the current study includes fathers and mothers simultaneously to investigate the associations between parental PS and adolescent DS. This study also examines the mediating effects of fathers’ and mothers’ individual overt and covert parental CCB in the relations (See Fig. 1).

During the home quarantine period, parental PS might become serious for parents in different family types and structures, particularly for single parents and separated/divorced/foster parents [57]. Previous studies [10, 28, 52] showed that additional stress might be bought to parents in these families that other parents typically have not encountered, which might increase parental PS and lead to disruptions of the coparenting relationship between parents. For example, previous research revealed that foster mothers with higher perceived parental PS also tended to have poorer coparenting relationships with their spouses [52]. Coparenting in these families might become vulnerable during the pandemic due to the dramatic changes in employment and childcare, such as working at home and closing schools and childcare centers [13, 16, 72]. A recent study reported social support, parental PS, and DS of 202 single mothers who have experienced divorce, separation, death, or have never married [57]. This study has revealed a low level of perceived social support and a high level of parental PS in single mothers for various reasons [57]. Hence, it needs to be further explored in future studies about parental PS and coparenting in different family types and structures, although this was not the central aim of this study (Fig. 1).

## Methods

### Procedure

#### Design

The target population consisted of a nationally representative sample of families, including fathers, mothers, and adolescents (10–18 years old) during the home quarantine period. Invitations were sent out to approximately 30 primary and secondary schools based on regional distribution across China, covering the regions of South China (e.g., Guangdong Province), Central China (e.g., Hubei Province), North China (e.g., Beijing City), East China (e.g., Shandong Province), Southwest China (e.g., Sichuan Province), and Northwest China (e.g., Ningxia Hui Autonomous Region). Data was collected through an e-questionnaire website, “Questionnaire Star,” between late April and early May 2020.

#### Informed consent process

First, informed consent, including survey objectives, the purpose of survey responses, and the estimated length of time, was sent by teachers to families willing to participate. Then, upon informed consent, participants were asked to fill out the questionnaire links. Our survey was anonymous; thus, personal information, including personal names and classes was not collected to avoid any self-reporting bias. Participants were free to withdraw from the research at any time. Last but not least, we also included our email address on the first page of the e-questionnaire in case the participating families required further support or assistance. The Research Ethical Committee of the first author’s institution approved the study.

#### Development and pre-testing

The questionnaires in our survey were all standardized in previous studies. The usability and technical functionality of the electronic questionnaire were checked and tested by two Ph.D. students before fielding the questionnaire.

#### Recruitment process

We did not make initial contact with our potential participants on the Internet. Our questionnaires were sent out to parents and adolescents with the assistance of the education department or schools. The survey was distributed through invitation instead of being advertised.

#### Survey administration

Teachers sent our participants the questionnaire links through the social networking app WeChat. An online informed consent statement outlining the purpose and procedure of this study and requesting consent was sent to all participants. Fathers, mothers, and adolescents were given the same 8-digit ID number, constituted by the last four digits of the father’s number plus the last four digits of the mother’s number. For example, the father’s cell phone number is 13637889323, and the mother’s is 15235732905. Hence, the 8-digit ID number of this family member is 93232905. In the survey, we repeatedly asked fathers, mothers, and adolescents in the same family to report their ID numbers to ensure they belonged to the same family.

#### Response rate

Regarding the analysis, the online survey software was programmed in advance so that participants were required to complete all the questionnaire items before submitting their answers. Therefore, there were no missing values in the final dataset in the submitted questionnaires. Participants with incorrect ID numbers (e.g., 9-digit or 4-digit numbers or wrong numbers) were considered inattentive. Moreover, our surveys were based on the unit of the two-parent family. Therefore, if we could not find the questionnaires of an individual’s family members using the same 8-digit ID number in the dataset, this participant would not be included in this analysis. The completed but non‐submitted and incomplete surveys were not recorded on the e-questionnaire website, “Questionnaire Star,” and thus could not be included in the analysis. There are 1162 families, including fathers, mothers, and one adolescent from 27 schools, who initially completed the questionnaire online. To ensure that our results were based on valid responses, one quality response item (i.e., “Please choose ‘4’”) was embedded throughout the surveys of fathers, mothers, and adolescents. After excluding inattentive respondents (including quality response items, other invalid answers, or matching problems), a total of 1031 families were retained in this study, resulting in a response rate of 88.7%.

### Participants

This study was a part of a national survey of Chinese families. The participants were families with adolescents at 27 primary and secondary schools based on regional distribution. Initially, 1162 families participated in the study. To ensure that our results were based on valid responses, we embedded one quality response item (i.e., “Please choose ‘4’”) throughout the surveys of fathers, mothers, and adolescents. After excluding inattentive respondents, 1031 families were retained in this study, resulting in a response rate of 88.7%. The father, mother, and one adolescent from each family participated in the present study. The mean ages of adolescents were 14.36 ± 2.30, ranging from 10 to 18 years old. The mean ages of fathers were 44.54 ± 4.48, ranging from 31 to 59 years old. The mean ages of mothers were 42.54 ± 4.26, ranging from 30 to 57 years old. The detailed demographic information of the participants is presented in Table 1.

**Table 1:** Demographic information of participants

Variables	Fathers		Mothers		Adolescents	
	N/M	%/SD	N/M	%/SD	N/M	%/SD
Age	44.70	4.56	42.83	4.23	14.36	2.30
Gender						
Boys					517	50.1%
Girls					514	49.9%
Only-child status						
The only child					597	57.9%
Non-only child					434	42.1%
SES	6.18	1.93	6.13	1.93		
Educational status						
Primary school	43	4.2%	61	5.9%		
Lower general secondary school	219	21.2%	239	23.2%		
Higher general secondary school	270	26.2%	256	24.8%		
Higher vocational education	175	17.0%	203	19.7%		
University education	324	31.4%	272	26.4%		

### Measures

#### Parental PS

Parental PS was measured by the Chinese version of the Parenting Stress Index-Short Form (C-PSI-SF), which consists of three subscales, namely parental stress, parent–child dysfunctional interaction, and difficult child [71], and was initially developed by Abidin in 1995 [1]. All items were measured on a 5-point Likert scale (from 1 = strongly agree to 5 = strongly disagree). Each subscale had 12 items, and the item scores were averaged. A higher score indicated a higher level of parenting stress a parent was experiencing. The C-PSI-SF has been confirmed to be reliable and valid in Chinese parents and is frequently used to assess parenting stress [36, 71]. In the present study, Cronbach’s alphas of mothers’ and fathers’ parenting stress were both 0.95.

#### Coparenting conflict behavior

Fathers’ and mothers’ coparenting behaviors were measured by the adapted Chinese version of the Coparenting Behavior Scale [35, 41, 75] reported by adolescents. This version includes 29 items of four dimensions [35, 75]. We used the conflict behavior dimension to represent overt CCB (conflict between mothers and fathers in the presence of the child, e.g., “My father argued with my mother in front of me”; 6 items) while using the disparaging behavior dimension to measure covert CCB (largely covert parent-to-child communication that reproaches or discredits the other parent, e.g., “My father commented negatively about my mother in front of me when she was absent”; 6 items). All items were measured on a 7-point Likert scale ranging from 1 (“absolutely never”) to 7 (“almost constantly”). Item scores were averaged in each dimension, and a higher score indicated a higher level of CCB. Adolescents reported fathers’ and mothers’ CCB. In the present study, Cronbach’s alpha for paternal CCB reported by adolescents was 0.89, and it was 0.93 for maternal CCB reported by adolescents.

#### Depressive symptoms

Child depressive symptoms were measured using the Zung Self-Rating Depression Scale [63, 76]. Of the 20 items, 10 were worded positively in relation to symptoms, and the other 10 were worded negatively. Items are presented as statements, and respondents indicate whether each symptom is true of themselves on a 4-point Likert scale from 1 to 4, “none or little of the time,” “some of the time,” “a good part of the time,” or “most of the time.” Ten of the items express negative experiences or symptoms (e.g., “I have crying spells or feel like it”), and 10 express positive experiences and are reverse scored (e.g., “My life is pretty full”), with total scores ranging from 20 to 80*.* The total score was averaged and used in the subsequent analysis. The reliability and validity of this scale for assessing depressive symptoms have been confirmed in Chinese adolescent populations [62]. The Cronbach’s alpha of SDS for adolescents in the current study was 0.89.

#### Subjective socioeconomic status

The subjective socioeconomic status (SES) scale was used to assess the participants’ subjective SES [46]. This scale was presented through a picture with a 10-grid ladder, ranging from 1 (lowest SES) to 10 (highest SES). Subjects were asked to choose the number that best represented the SES of their household.

#### Control variables

We collected the parents’ ages, educational levels, and subjective SES in both the parents’ questionnaires. In the children’s questionnaires, we collected their age, gender (male or female), and only-child status (only one child or more than one child). All of these variables were controlled in the subsequent analysis.

### Data analysis

Descriptive statistics and Pearson correlational analysis were conducted using SPSS 26.0. Structural equation modeling (SEM) was performed to examine the mediation model. Bootstrap analysis with 2000 replicates and 95% confidence intervals were used to examine the significance of the mediation effects. The characteristics of parents (age, educational level, and SES) and adolescents (age, gender, and only-child status) were treated as control variables in the analysis.

## Results

### Preliminary analysis

Table 2 presents the study variables’ means, standard deviations, and Pearson correlations.

**Table 2:** Intercorrelations, means, and standard deviations for study variables

Variable	1	2	3	4	5	6	7	8	9	10
1 Age	1									
2 Gender	− 0.027	1								
3 OC	− 0.120**	0.046	1							
4 PPS	0.023	− 0.044	0.126**	1						
5 MPS	0.017	− 0.093**	0.158**	0.492**	1					
6 POCCB	0.078*	− 0.009	0.045	0.226**	0.281**	1				
7 PCCCB	0.063*	− 0.054	0.035	0.197**	0.258**	0.729**	1			
8MOCCB	0.101**	− 0.009	0.036	0.188**	0.263**	0.780**	0.715**	1		
9 MCCCB	0.072*	− 0.033	0.044	0.180**	0.272**	0.664**	0.828**	0.799**	1	
10 ADS	0.127**	0.074*	0.064*	0.286**	0.345**	0.401**	0.320**	0.383**	0.354**	1
M				2.132	2.157	2.215	1.664	2.020	1.706	1.653
SD				0.699	0.715	1.345	1.119	1.313	1.151	0.491

*PPS* paternal parenting stress, *MPS* maternal parenting stress, *POCCB* paternal overt coparenting conflict behavior, *PCCCB* paternal covert coparenting conflict behavior, *MOCCB* maternal overt coparenting conflict behavior, *MCCCB* maternal covert coparenting conflict behavior, *ADS* adolescent depressive symptoms

*p < 0.05; **p < 0.01; ***p < 0.001

### Testing the mediation effect model

**Fig. 2:**
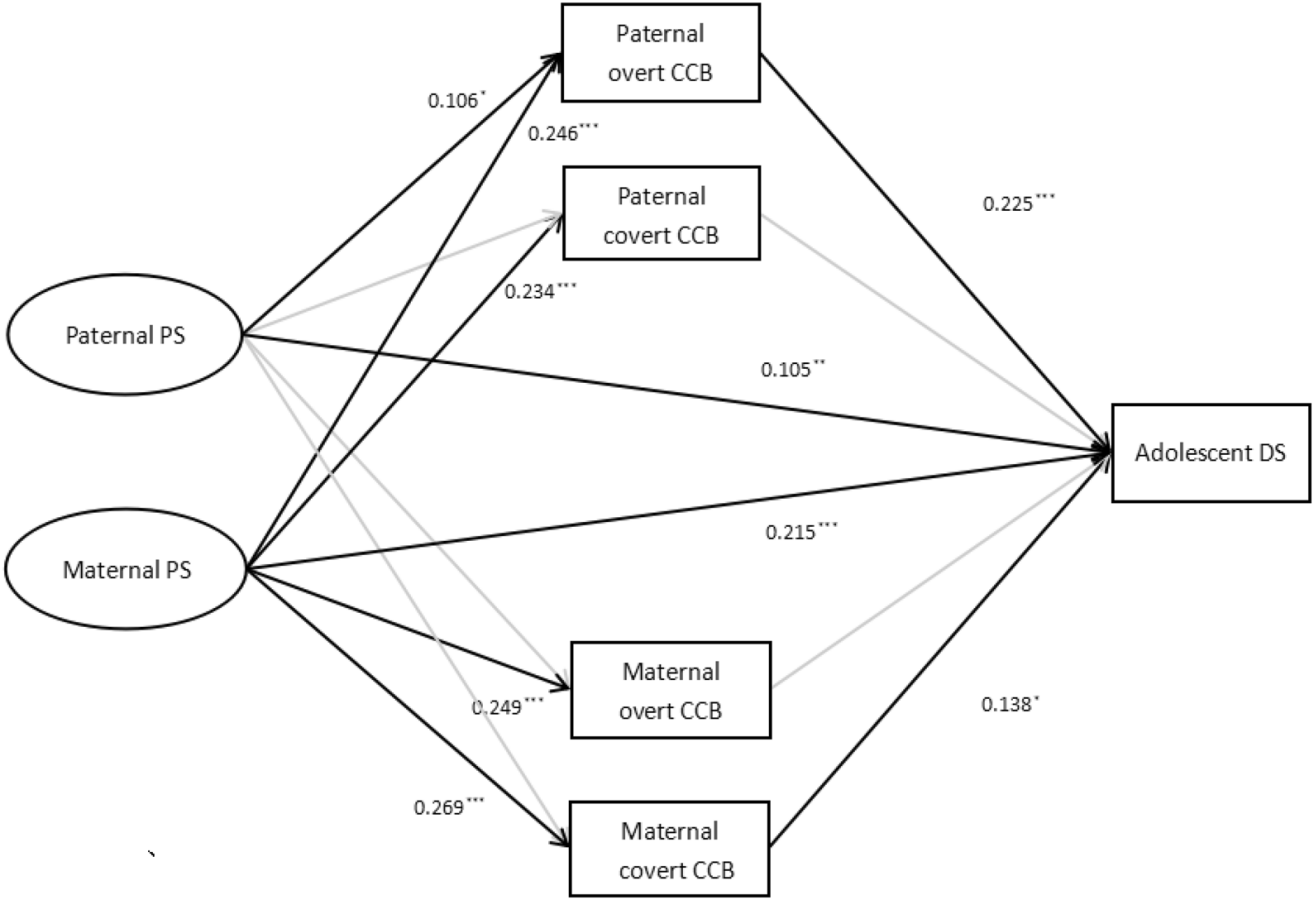
The mediation model

Figure 2 presents the standardized coefficients of the mediation effect model. The model showed acceptable fit indices (χ^2^/*df* = 4.34, RMSEA = 0.057, CFI = 0.956, TLI = 0.942, SRMR = 0.077). As Table 3 illustrates, the 95% bootstrap confidence intervals of the mediating effects of paternal overt and maternal covert CCB between the relationship of maternal PS and adolescent DS did not include zero. Thus, these two mediating effects were statistically significant.

**Table 3:** The bootstrap confidence interval and effect size of the overall mediation model

Mediation paths	Estimate	P	95% CI
PPS → Paternal overt CCB → ADS	0.024	0.065	[− 0.001, 0.049]
PPS → Paternal covert CCB → ADS	− 0.008	0.324	[− 0.023, 0.007]
PPS → Maternal overt CCB → ADS	0.005	0.464	[− 0.008, 0.018]
PPS → Maternal covert CCB → ADS	0.006	0.444	[− 0.010, 0.022]
**MPS → Paternal overt CCB → ADS**	**0.055**	**0.004**	**[0.017, 0.094]**
MPS → Paternal covert CCB → ADS	− 0.022	0.172	[− 0.053, 0.010]
MPS → Maternal overt CCB → ADS	0.018	0.326	[− 0.018, 0.053]
**MPS → Maternal covert CCB → ADS**	**0.037**	**0.043**	**[0.001, 0.073]**

Only the significant paths were portrayed as bold

*PPS* paternal parenting stress, *MPS* maternal parenting stress, *POCCB* paternal overt coparenting conflict behavior, *PCCCB* paternal covert coparenting conflict behavior, *MOCCB* maternal overt coparenting conflict behavior, *MCCCB* maternal covert coparenting conflict behavior, *ADS* adolescent depressive symptoms

*p < 0.05; **p < 0.01; ***p < 0.001

## Discussion

The current study adopted an interdependent approach to construct a mediation model that examined the effects of parental PS on adolescent DS through the mediating effects of overt and covert parental CCB in China. Paternal PS was related to adolescent DS through the mediating effect of paternal overt CCB. Maternal PS was related to adolescent DS through the mediating effects of paternal overt CCB and maternal covert CCB. Overall, maternal PS exerted a more substantial influence on adolescent DS than paternal PS. The study supplemented understanding of the relationships and mediating mechanisms between parental PS and adolescent DS, which promotes the targeted family intervention programs.

### The relationship between parental PS and adolescent DS

Our results showed that parental PS was, directly and indirectly, related to adolescent DS. Consistent with Abidin’s parental stress model, parenting stress was recognized as a critical determinant of child DS [1, 2]. It was particularly important for us to assess parental PS during the COVID-19 quarantine period because parents experienced increasing levels of parental PS. Parental PS may cultivate a sense of uncontrollability and trigger associated negative emotional experiences in parents, which may be transmitted to children and result in children’s DS [6, 14, 48, 54, 60].

It is of note that maternal PS was also directly related to adolescent DS, while paternal PS was not. This may reflect the phenomenon that mothers are usually the primary caregiver in Chinese families; they usually shoulder a greater share of household responsibilities and dedicate more time to child-rearing in the family [33, 70]. Mothers are also less likely to reduce time spent on child rearing than fathers due to other factors that may result in higher PS in mothers [53, 70]. In this case, maternal PS may be more likely than paternal PS to transmit to adolescents and exacerbate their DS [14, 45].

### The mediating role of parental CCB

Consistent with the parental stress model [1, 2], our study supported that parental CCB constitutes a crucial mediating mechanism in the relationship between parental PS and adolescent DS. Stressed parents have tended to respond to parenting stimuli negatively due to excessive anxiety and worry [11], thus, they may not be able to provide an optimal interpersonal environment for children [11]. With elevated parental PS, the parent may become dissatisfied with the other parent, demonstrate disrespectful attitudes, blame the other parent’s mistakes, and complain to the child. When struggling with parental PS, parents may be more likely to become involved in interparental conflicts due to energy depletion. Exposure to coparenting conflicts during daily interactions contributes to the risk of developing DS in adolescents.

Only fathers’ overt CCB and mothers’ covert CCB demonstrated significant mediating effects on the relationship between parental PS and adolescent DS. This may be due to the tendency of children to hold different expectations and interpretations of their fathers’ and mothers’ behaviors [34]. As adolescents grow older, they may become more sensitive to and less tolerant of fathers’ stressed behaviors [74]. Since families may expect mothers, as the primary caregiver, to take the main responsibility of parenting at home, mothers may have more opportunities to develop a stable emotional bond with children and show disparaging behavior toward them [38]. If the mother undermines the father when alone with the child, the child’s consistent and positive view of the family may be negatively influenced. In particular, the child may feel distant toward both parents and experience more negative emotions when the mother disparages the father’s absence [55, 74].

It is mainly maternal PS that influences adolescent DS through paternal and maternal CCB. In our study, maternal PS was more strongly related to paternal overt and covert CCB than paternal PS itself. This may be explained by the fathering vulnerability hypothesis [49]. The father’s parenting is more likely to be affected by other factors than the mother’s, thus demonstrating higher susceptibility to external stressors [12, 49]. This may be because fathers are less clearly defined by social conventions than mothers, which makes fathering more vulnerable to external influences [12, 49]. Our study also found that maternal PS could influence adolescent DS through paternal CCB, but paternal PS could not influence adolescent DS through the mediating effects of maternal CCB.

Although we have included parents’ age [57], SES [58], and educational levels as controlling variables, other critical contextual factors that may influence parental PS and coparenting conflicts, such as employment, work status, and work-and-family conflicts [23] during the lockdown period, have not been controlled. Previous research has indicated the importance of a variety of contextual factors (e.g., work hours and conflict of work and family) for parental PS [33, 45] and coparenting conflicts [53]. For example, a recent study [23] revealed that the feasibility of remote working acted as a key influential factor for parental PS among the pandemic-related contextual factors. The work-family balance may constitute a conspicuous challenge for parents during the lockdown that may hamper parental PS, which might impact coparenting [23].

Moreover, the status of work from home might influence the gendered division of housework and childcare and thus differentially impact parental PS for fathers and mothers. A recent study [16] found that when both parents worked at home, although fathers’ family involvement increased, this would not change the gendered phenomenon of household work, as mothers also tended to contribute more time and energy to household activities. When neither parent or only the mother worked at home, mothers might also become the main force in charge of the extra caring and schooling of children [16], remaining gendered responsibility. Another recent study [72] also found that mothers have carried a heavier load than fathers in childcare during the pandemic, even while they are still working. However, it seems that mothers’ working status only has a limited influence. Under stressful circumstances, mothers tended to reduce their working hours to balance the childcare demand divisions. Moreover, working mothers are more likely to transition out of employment due to childrearing. Researchers [13] have revealed that compared to the father, a working mother is more than four times more likely to decrease their work hours for childcare, indicating the challenges of the pandemic to mothers’ employment and the unequal domestic division of labor. Although these studies [13, 16, 72] did not reveal the direct relationship between work status and parenting PS and coparenting, they indicated the difference in paternal and maternal PS and the increased possibility of coparenting conflicts due to gendered divisions of housework. Therefore, future studies might examine work-related variables during the pandemic and control them in the analysis to explore the associations between parental PS, parental CCB, and adolescent DS.

Moreover, as a limitation of our study, other risk factors have not been included in our study. Indeed, parents and adolescents have encountered contextual, family, interpersonal, individual, and other multiple factors during the pandemic. Researchers [22] have posited that families have been exposed to a series of stressors, such as work and financial disruptions, social isolation, school and childcare closures, and shelter-in-place isolation brought by the pandemic in a comprehensive model. These multiple risk factors would lead to disruptions of parents (stress, financial strain, and mental health), which would then affect individual parenting and coparenting quality [22], and finally influence child adjustment (mental health, emotional security, and behavior problems). Empirical studies have also suggested the various influences of contextual factors such as “work status” [23] and “SES” [58], family factors such as “household chaos” [58], interpersonal factors such as “marital status” [30], and “individual factors” [15, 30, 64] on parental PS, coparenting, and adolescent adjustments. Therefore, future research might explore the influences of other risk or protective factors on parental PS, coparenting, and adolescent adjustments, or the associations after taking multiple influential factors into account.

Finally, this study was conducted during the lockdown period in China. It should be noted that some empirical studies have pointed out the detrimental effects of restricted activities on families [47, 58]. For example, a study conducted during the lockdown [58] showed that the lockdown could increase parental PS levels to some extent, and it is pretty stressful for parents to achieve a balance between life, work, and childcare in the absence of other social help during this particular period [58]. A recent study [22] found a large deterioration in the magnitude of within-individual change in parent and child mental health in the first months of the pandemic compared to before. Moreover, researchers [22] pointed out that coparenting and individual parenting quality also tended to decline during this period. Based on the results, researchers have emphasized the need for intervention programs to provide more support and guidance and thus prevent families from potential “scarring,” which could be regarded as a prolonged and intertwined individual and family problems. Another study [47] recorded changes of parents over the first 1.5 years of the pandemic and compared measurement results before and within two mandatory lockdowns during the pandemic. The findings showed that parents displayed elevated DS, reduced well-being, and poor physical health; poor cohesion in family ties and more chaos, but no obvious change in the parent–child relationship and parenting practices. Importantly, partner support as a buffer could protect family functioning from the adverse effects of the lockdown.

Overall, elevated mental health problems and decreased family functioning emerged in families faced with multiple risk factors [7, 8] during the pandemic, but some families could also achieve resilient outcomes by adapting to these changes [18, 47, 50]. For example, one study revealed no change in couple relationship satisfaction from shortly before to shortly after the pandemic [69]. Moreover, another study adopted mix-methods to investigate the influence of the pandemic on adolescent parents [3]. While the qualitative analysis of 21 adolescent parents revealed more adverse effects of the pandemic, such as economic and health stress [3], the quantitative analysis showed that the pandemic-period cohort also demonstrated fewer DS, parental PS, more positive coparenting communication, and conflict management than the similar pre-pandemic cohort counterparts from the same school [3]. By adopting mixed methods in a sample with relatively good SES, researchers [18] found that families still encountered challenging struggles in various life domains from May to July 2020, indicating significant associations between the pandemic’s daily impact and elevated psychological distress for children and parents. However, some families still exhibited resilience and achieved certain levels of positive outcomes. Some resilience factors, such as family routines, family relationships of high quality (marital, parent–child, and coparenting), and constructive problem-solving [40, 50] could help families better respond and adapt to challenges during stressful times. Intervention programs should consider resilient factors that could mitigate and help families recover from the adverse effects.

Nonetheless, this study also contributed to knowledge about family adjustments during the pandemic. The current study demonstrated the need to simultaneously distinguish and investigate fathers’ and mothers’ parenting-related variables. Within the parental stress model framework, we tried to illuminate specific mechanisms whereby parental PS would flow to adolescent DS. The results revealed that the parent’s gender and individual PS interacted to affect adolescent DS. Maternal PS exerted a more substantial effect on adolescent DS than paternal PS. Maternal PS could also spill over and cross over into maternal covert CCB and paternal overt CCB, which would then influence adolescent DS. However, paternal PS could only spill over into the father’s overt CCB and influence adolescent DS.

## Limitations

Some limitations need to be acknowledged. First, this study used a cross-sectional design to collect data, which precluded us from determining the causal relationships between parental PS, CCB, and child DS. There may also be an overestimation of the effect size. Second, this study only investigated the mediating effects of parental CCB between the relationships of parental PS and child DS. Whether the characteristics of adolescents (e.g., emotion regulation) or other family relationship factors (e.g., parent–child relationship) play a role in the relationships remains unclear.

## Implications

Despite the limitations above, the current study provides some implications. First, considering the strong effects of parental PS on adolescent DS, interventions aimed at reducing parental PS may be an important direction. Second, taking steps to minimize father–mother conflicts over parenting may be effective for intervening with adolescent DS. Third, the results revealed that fathers’ overt CCB and mothers’ covert CCB mediated the relationships between parental PS and adolescent DS. Therefore, experts could offer targeted suggestions to reduce the likelihood of fathers becoming involved in conflicts with mothers and encourage mothers to make fewer covert disparaging remarks about fathers in front of the child when the father is absent.

## Conclusion

Parental PS is related to adolescent DS through the mediating effects of paternal overt CCB and maternal covert CCB. Overall, maternal PS exerts a more substantial influence on adolescent DS than paternal PS.

## Implications and contributions of this manuscript

This study clarifies the relationship between paternal and maternal parenting stress and adolescent depressive symptoms. It also illuminates different mechanisms whereby paternal and maternal parenting stress influences adolescent depressive symptoms, highlighting the important mediating effects of paternal overt and maternal covert coparenting conflict behaviors. This could provide targeted suggestions for intervention programs for families with adolescents.

## Data Availability

The datasets generated and/or analyzed during the current study are available from the corresponding author upon reasonable request.
